# Editorial: Medicinal chemistry for neglected tropical diseases using *in-vitro*, *in-vivo* and *in silico* approaches

**DOI:** 10.3389/fchem.2025.1689034

**Published:** 2025-09-01

**Authors:** Suraj N. Mali, Essa M. Saied, Cleydson Breno Rodrigues dos Santos, Jorddy Neves Cruz

**Affiliations:** 1 School of Pharmacy, DY Patil University, Navi Mumbai, Maharashtra, India; 2 Humboldt University of Berlin, Berlin, Germany; 3 Federal University of Amapá, Macapá, Amapá, Brazil; 4 Federal University of Pará, Belém, Pará, Brazil

**Keywords:** drugs, bioactive componds, natural products, new therapies, pharmacology

Neglected tropical diseases continue to pose a major challenge to global health, disproportionately affecting resource-limited regions and vulnerable populations. Despite their epidemiological importance, the development of innovative therapies remains restricted due to limited investment and the biological complexity of the pathogens involved. In this context, medicinal chemistry plays a central role in the search for novel pharmacological solutions, as it enables the integration of *in vitro*, *in vivo*, and *in silico* approaches for drug discovery (Ferreira L. L. G. et al., 2022; Ferreira O. O. et al., 2022).

The study by Maliyakkal et al. presents a two-dimensional QSAR model based on machine learning to accelerate drug discovery against *Trypanosoma cruzi*, the etiological agent of Chagas disease, which still lacks effective therapies. Using a dataset of 1,183 inhibitors, predictive models were developed and validated, with the artificial neural network (ANN-QSAR) showing the highest accuracy. Virtual screening identified 12 promising compounds, with F6609-0134 emerging as the most relevant candidate, as further confirmed by molecular docking, molecular dynamics simulations, and free energy calculations Maliyakkal et al.

Zanthoxylum rhoifolium Lam., traditionally used in folk medicine as an antiparasitic agent, demonstrated promising results against *Leishmania amazonensis*. The ethanolic extract and its fractions, particularly the neutral fraction, revealed chromatographic profiles suggestive of alkaloids, with chelerythrine being isolated and identified. In biological assays, the extract and fractions exhibited dose-dependent leishmanicidal activity, with the neutral fraction showing high selectivity and low cytotoxicity in VERO cells. Moreover, this fraction enhanced cell proliferation and promoted wound-healing effects, reinforcing the therapeutic potential of this species both in parasite control and tissue regeneration (Correa-Barbosa et al.).

Tuberculosis remains a serious global health problem, further complicated by the emergence of drug-resistant *Mycobacterium tuberculosis* strains. In this context, one study explored the therapeutic target PknG, a kinase essential for bacillary survival in macrophages, using multiple computational approaches to screen 460,000 molecules. Seven high-affinity compounds were identified, among which chromene glycoside (Hit 1) displayed the most favorable pharmacokinetic profile, low toxicity, and stability confirmed by molecular dynamics simulations. DFT analyses further supported its superior electronic properties compared to the reference ligand, consolidating it as a promising candidate for the development of new drugs against resistant TB strains (Alruwaili et al.).

The growing threat of resistant tuberculosis, including MDR-TB and XDR-TB, underscores the urgent need to identify new therapeutic targets against *M. tuberculosis*. In this *in silico* study, microbial natural products from the NPAtlas database were screened as potential inhibitors of DNA gyrase B, an enzyme essential for bacillary replication. Twelve initial compounds were identified and further refined through ADME-T evaluation, pharmacophore modeling, and molecular dynamics simulations. Among them, 1-Hydroxy-D-788-7, an anthracycline derivative, stood out for its higher affinity and binding stability, along with Erythrin and Pyrindolol K2. The findings highlight 1-Hydroxy-D-788-7 as the most promising candidate, reinforcing the potential of microbial natural products in the discovery of new agents against resistant TB strains, although further experimental studies are required to validate its therapeutic efficacy (Elsaman et al.).

Dengue continues to represent a global challenge due to the wide spread of its vectors and the limitations of available therapies and vaccines, emphasizing the need for new antiviral agents. Hanson et al. applied integrative approaches combining machine learning, molecular docking, and molecular dynamics simulations to identify potential inhibitors of the dengue virus NS2B/NS3 protease, an enzyme essential for viral replication. From over 21,000 compounds screened, the logistic regression model achieved 94% accuracy, highlighting four lead molecules (ZINC38628344, ZINC95485940, 2′,4′-dihydroxychalcone, and ZINC14441502), all showing high binding affinity and stable interactions with the catalytic triad of the enzyme. These compounds also exhibited favorable pharmacokinetic properties and low predicted toxicity, demonstrating their potential as safe and promising candidates for the development of novel anti-dengue therapeutics (Hanson et al.).

The study on *Ruta montana* L., a medicinal plant from the Middle Atlas region of Morocco, provided a detailed phytochemical characterization and a comprehensive evaluation of its biological properties. The essential oil, rich in 2-undecanone (81.16%), exhibited strong antimicrobial activity, particularly against fungi, along with analgesic and anti-inflammatory effects comparable to reference drugs. Phenolic extracts, especially those containing rosmarinic acid derivatives and quercitrin, showed pronounced antioxidant activity. Toxicological assays indicated relative safety at controlled doses, although mild biochemical alterations suggest caution at higher concentrations. These findings highlight the potential of this species as a natural source of antimicrobial, antioxidant, and anti-inflammatory agents (El Ouardi et al.).

The study by Zhang et al. investigated the phenolic compounds present in Bairui, a traditional preparation derived from *Thesium chinense* Turcz., identifying five major compounds: methyl-*p*-hydroxycinnamate, vanillin, kaempferol, isorhamnetin-3-*O*-glucoside, and astragalin. Notably, three of these compounds were reported for the first time in this formulation, expanding knowledge of its chemical composition. Co-culture assays demonstrated that isorhamnetin-3-*O*-glucoside promoted the proliferation of Chinese hamster ovary cells, human umbilical cord blood NK cells, and mesenchymal stem cells, while vanillin and astragalin also showed positive effects on immune cell expansion at specific concentrations. Conversely, some compounds displayed selective inhibitory effects on peripheral NK cells (Zhang et al.).

Research on hydroethanolic leaf extracts of *Ficus carica* L. from eastern Morocco, conducted by Tikent et al., revealed a rich profile of bioactive compounds, particularly polyphenols, flavonoids, vitamin C, and condensed tannins, associated with strong antioxidant activity confirmed by multiple assays (DPPH, β-carotene, ABTS, and TAC). Additionally, the extracts showed significant antimicrobial activity against both Gram-positive and Gram-negative bacteria, as well as *Candida albicans*. *In vitro* assays also revealed selective cytotoxicity against three breast cancer cell lines (MCF-7, MDA-MB-231, and MDA-MB-436), suggesting antitumor potential. *In silico* studies further indicated possible mechanisms of interaction between the metabolites and bacterial, fungal, and tumor targets, reinforcing their pharmacological versatility (Tikent et al.).

The study on *Aspidosperma nitidum* conducted by Brígido et al. provided important insights into its therapeutic potential against cutaneous leishmaniasis, demonstrating both antiparasitic activity and immunomodulatory effects in BALB/c mice infected with *L. amazonensis*. Ethanolic extracts and their alkaloid-rich fractions contained compounds such as corynantheol, yohimbine, and dihydrocorynantheol, identified by mass spectrometry, and confirmed *in silico* to interact stably with catalytic residues of trypanothione reductase, a validated leishmanicidal drug target. *In vivo*, treatment significantly reduced lesion progression and splenic parasite load in a dose-dependent manner, achieving up to 42.5% reduction. Furthermore, immune modulation was observed, with decreased IL-10 expression and increased IFN-γ production, promoting a protective host response (Brígido et al.).
